# Cortical Oscillations during Gait: Wouldn’t Walking Be So Automatic?

**DOI:** 10.3390/brainsci10020090

**Published:** 2020-02-09

**Authors:** Arnaud Delval, Madli Bayot, Luc Defebvre, Kathy Dujardin

**Affiliations:** 1 UMR-S1172, Lille Neuroscience & Cognition, Inserm, University Lille, 59000 Lille, France; madli.bayot@gmail.com (M.B.); luc.defebvre@chru-lille.fr (L.D.); kathy.dujardin@univ-lille.fr (K.D.); 2 Clinical Neurophysiology Department, CHU Lille, 59000 Lille, France; 3 Movement Disorders Department, CHU Lille, 59000 Lille, France

**Keywords:** Gait, EEG, oscillations

## Abstract

Gait is often considered as an automatic movement but cortical control seems necessary to adapt gait pattern with environmental constraints. In order to study cortical activity during real locomotion, electroencephalography (EEG) appears to be particularly appropriate. It is now possible to record changes in cortical neural synchronization/desynchronization during gait. Studying gait initiation is also of particular interest because it implies motor and cognitive cortical control to adequately perform a step. Time-frequency analysis enables to study induced changes in EEG activity in different frequency bands. Such analysis reflects cortical activity implied in stabilized gait control but also in more challenging tasks (obstacle crossing, changes in speed, dual tasks…). These spectral patterns are directly influenced by the walking context but, when analyzing gait with a more demanding attentional task, cortical areas other than the sensorimotor cortex (prefrontal, posterior parietal cortex, etc.) seem specifically implied. While the muscular activity of legs and cortical activity are coupled, the precise role of the motor cortex to control the level of muscular contraction according to the gait task remains debated. The decoding of this brain activity is a necessary step to build valid brain–computer interfaces able to generate gait artificially.

## 1. Introduction

Gait control in natural environments (e.g., passing through a doorway, stepping an obstacle, initiating gait,…) can only be achieved on the basis of proprioception, visual and vestibular signals, and implies cognitive control [1]. These processes occur mainly at cortical and cerebellar levels and compose the voluntary aspect of walking. Measuring brain activity during gait with sufficient temporal resolution can help to determine which brain areas are involved in motor behavior control. Electroencephalography (EEG) presents better temporal resolution than other brain imaging methods to record cortical activation during gait. Indeed, gait is often considered as an automatic activity which can be modified by cortical activations in certain circumstances that necessitate adaptation of gait pattern [2]. Gait is actually composed of repetitive stereotyped gait cycles. Each cycle consists of two phases: following foot contact, the leg is on the ground supporting the gravitational load of the body and propelling the body forwards. This first phase is called the stance phase, with two periods of double contact. Then, during the swing phase, the leg is lifted from the ground by the muscles and is moved against its inertial load. The changeover of relatively similar right and left cycles occurs rhythmically. Animal studies in decerebrate cats suggest that central pattern generators which are responsible for rhythmic movements are located in the spinal cord. Indeed, the spinal cord itself contains neural circuits that, when activated, can coordinate the different muscles to produce locomotor movements [3]. Adaptations of this motor program are necessary during gait initiation, stops, turns, obstacles, or following changes in the environment. A central network generates essential features of the motor pattern and sensory feedback signals control the system. In mammals, locomotor regions situated in the brainstem can directly activate central pattern generators located in the medulla and are under the control of the basal ganglia, cerebellum and cortex [3]. There is now large evidence of the role of the cerebral cortex in gait control [1]. In fact, numerous interactions exist between motor control of gait and cognition. Some of these interactions, often defined as dual-task interactions, are common in daily life, and involve common situations such as walking while simultaneously talking, texting on a cell phone or thinking about one’s shopping list [4,5]. Dual-task walking abilities in humans are at least in part under the control of cortical prefrontal areas [6], but other cortical and subcortical regions are also involved [5]. 

**Figure 1:**
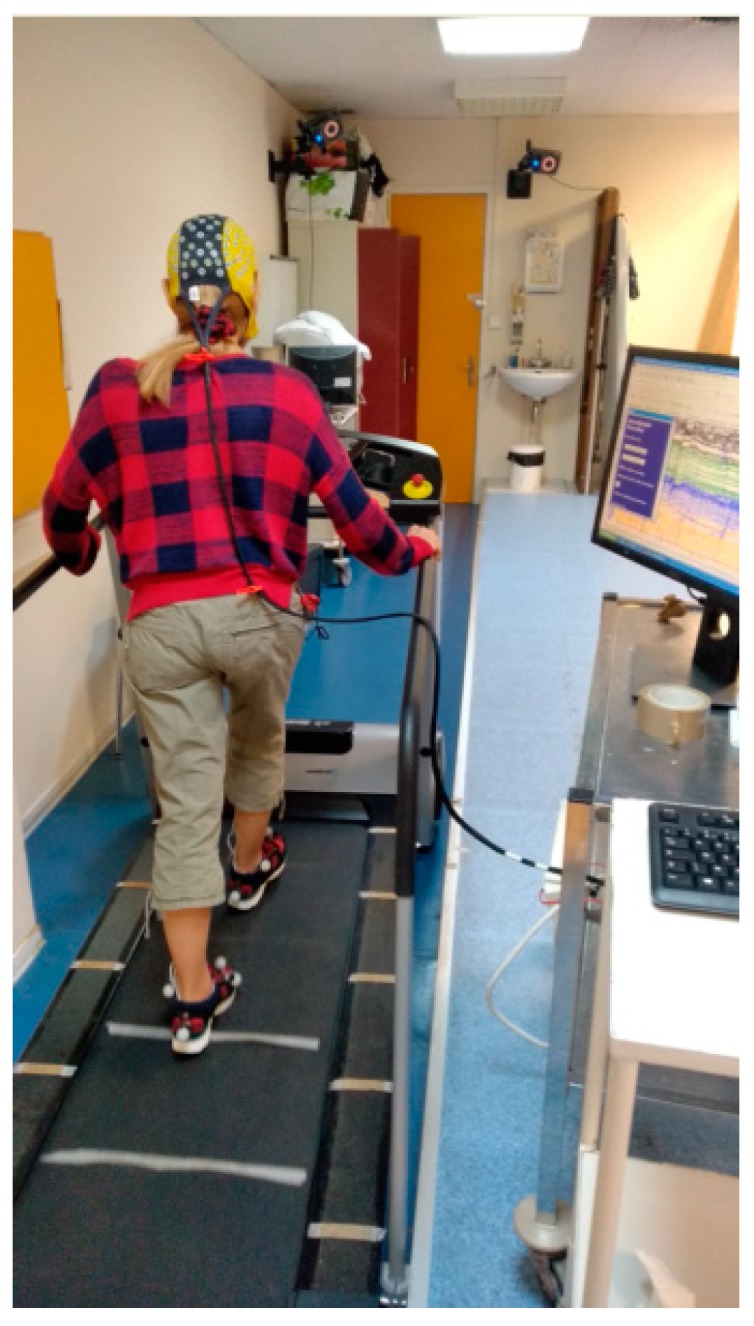
EEG recording during treadmill walking in a healthy subject. The EEG system is synchronized with a video-based motion analysis system (retroreflective markers on both feet) that allows recording of the different events (foot off and strike) characterizing gait. Cortical activity was recorded with an Ag/AgCl 128-scalp-electrode cap (Waveguard®, ANT Neuro, Enschede, The Netherlands). Data were acquired with ASA^TM^ software (ANT Neuro).

In order to study cortical activity during real locomotion, EEG appears to be particularly appropriate. Indeed, overall EEG devices have a compact size, are relatively low-cost and easily available. EEG is a non-invasive brain imaging modality and allows a direct assessment of neural activation with a high temporal resolution (in the order of one millisecond). One of the strengths of EEG is thus the possibility to assess brain functioning during online walking and to correlate cortical activation with gait measures given by other devices (video-based motion analysis systems, force insoles, inertial sensors, electromyogram,…) (Figure 1). By comparison, in the context of positron emission tomography (PET) or single photon emission computed tomography (SPECT), metabolic changes occur several minutes after the injection according to the marker used. Spatial resolution of EEG is also better than functional near-infrared spectroscopy. However, PET and SPECT scans present a higher spatial resolution than EEG, while functional magnetic resonance imaging (fMRI) is associated with still better spatial accuracy despite the low temporal resolution related to this neuroimaging modality due to the slowness of changes in blood flow following fast changes in electrical neuronal activity. The main limitation of the use of fMRI, PET, and SPECT scans for analyzing gait-related neural activation remains the required head immobility of the subjects.

In this narrative review, after some methodological considerations, we will mainly focus on recent literature regarding EEG spectral changes during gait initiation and stable gait in healthy subjects, restricting this paper to cortical activity. Most recent studies have investigated cortical oscillations while some of them have also analyzed event-related potentials (ERPs). However, the latter were not within the scope of this paper. Spectral analyses promote a better understanding of the neurophysiology of gait and the role of oscillations in the adaptation of gait to the environment.

## 2. Methodological Considerations

**Figure 2:**
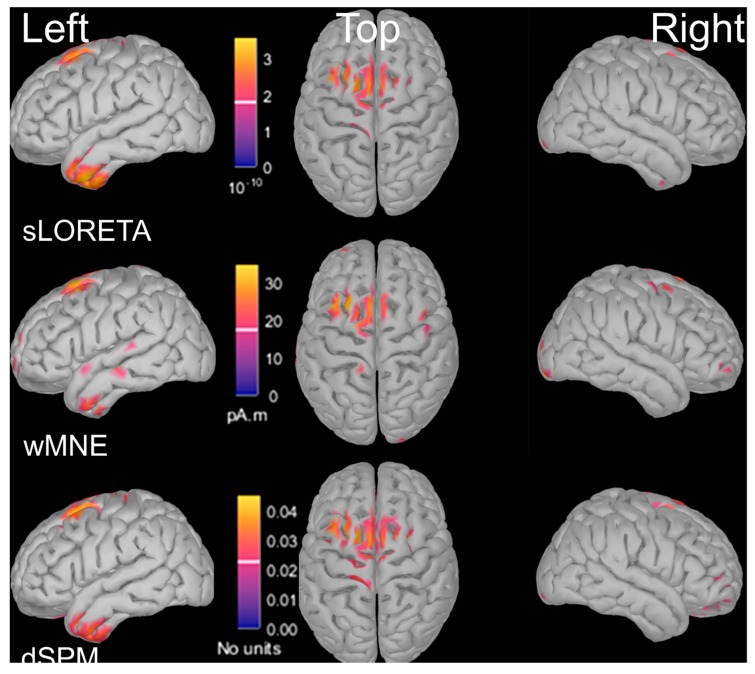
Source localization of EEG signal (4–30 Hz) during gait initiation in a paradigm using cues and flankers (task described in [16]). The signal was averaged over a time interval between 200 and 0 ms before the onset of the anticipatory postural adjustments. Three different methods for source localization were applied on the same EEG dataset (128 electrodes). All three methods showed sources in premotor cortex, supplementary motor area and primary motor cortex. Only sLORETA and dSPM showed sources on left temporal lobe but not wMNE.

Until recently, several concerns limited the use of EEG during real locomotion. Firstly, spatial resolution of EEG is low compared to PET, SPECT or MRI. Source localization from scalp EEG signals can be used to improve spatial resolution at the cortical level. Sources of EEG activity can be estimated by solving the inverse problem (i.e., identifying the location and calculating the amplitude and orientation of the neural sources that are responsible for the measured EEG data, based on these scalp EEG recordings). These characteristics of a source are adjusted in order to obtain a best fit between the recorded EEG signal and the calculated potentials produced by the source [7]. However, the main limit of this approach is that signals recorded by scalp electrodes mostly result from post-synaptic potentials of cortical neurons or synchronized activity of cortical neurons with deep sources (subcortical nuclei). There is still some disagreement among scientists about the possibility for EEG to record deep sources that are not synchronized with cortical activity [8]. Indeed, source analysis localized the irritative zone in patients suffering from epilepsy with high sensitivity and specificity if EEG signals were recorded with a large number of electrodes (128–256 channels) and if individual MRI was used as head model [9]. Other studies confirmed that a large number of electrodes is necessary to adequately solve the inverse problem (32 electrodes is not enough [10]), for example for a cognitive task (picture naming) [11]. However, the sources in these examples are cortical and, for some authors, subcortical signals are much weaker than cortical activity and deeper sources can be associated with distributed cortical activity [12]. Moreover, although the obvious differentiation between dipolar models with their a-priori assumed fixed number of dipoles and distributed source imaging techniques, the diversity of methods to solve the inverse problem as well as the difficulty of obtaining evidence about the true location of the sources makes it difficult to give any guidelines for the best method to choose. An example of different results of source localization methods during gait initiation is provided in Figure 2. Intracerebral recordings of local field potentials with deep brain stimulation electrodes can also be used to explore profound sources [13,14,15]. Unfortunately, they can only be performed in patients that require deep brain stimulation to reduce their symptoms. 

Secondly, artefacts caused by movement and muscle activity contaminate EEG signals. Of course, adequate filtering, bad channel repair by interpolation (flat electrodes or with high-amplitude noise), and the selection of epochs uncontaminated by obvious artefacts must be performed [17]. Another possible pre-processing step aims at removing transient, non-biological, large-amplitude noise/artefacts (e.g., abrupt impedance changes due to headset motions) using a non-stationary method based on sliding window principal component analysis: the Artifact Subspace Reconstruction (ASR) method [18]. Despite these essential first steps, two studies [19,20] investigated movement-related artefacts in EEG recordings and found contamination of the EEG data at frequencies from 1 to 150 Hz. As EEG signal frequencies investigated during walking include theta (4–7 Hz), alpha (8–12 Hz), beta 1 (13–20 Hz) beta 2 (20–30 Hz) and gamma (> 30 Hz) bands, these motion artefacts are taken into account in the analysis of cortical activity during gait and should be removed before considering motor-related changes in a power frequency band. 

Currently, the most frequently performed method to remove movement-related artefacts used independent component analysis (ICA) [21]. In this context, EEG is assumed to be a linear mixture of non-Gaussian and statistically independent source components that can be separated via ICA, visually examined, and classified as artefacts or EEG signal components [22]. Therefore, ICA allows to identify EEG sources, regardless of their localization. Once the artefact components have been identified, they can be removed. The remaining EEG signal components can be projected back to the original electrode space. This procedure yields the reconstruction of an artefact-free EEG signal. The number of independent components is equal to the rank of the matrix storing the original EEG signals (i.e., the total number of channels minus the amount of interpolated electrodes). We should note that, in reality, the effective number of statistically independent signals contributing to the scalp EEG is generally unknown. Although some sources correspond to obvious artefacts (e.g., eye blinks, horizontal and vertical eye movements), it is often difficult to determine with certainty whether the component represents cortical signal or not. Localization of the source on a scalp map, the component time course, the component activity power spectrum and an image of collected single-trial data epochs are crucial for identifying the nature of the considered independent component. It has also been proposed to use image processing algorithms on independent components in order to automatically reject EEG artefacts [23]. More generally, researchers can now use (semi-)automated EEG independent component classifiers, including *ICLabel* that proved the best classification accuracy and computational efficiency [24]. These classifiers that were highly trained on components labelled by experts of the field help to eliminate EEG artefacts. Other algorithms that model independent components as equivalent current dipoles can be used to localize neural sources (DIPFIT) [25]. A recent study tested the ability of ICA combined with DIPFIT as a source localization algorithm in order to remove EEG artefacts during treadmill walking (cortical signal blocked by a silicon cap) [26]. ICA and dipole fitting accurately localized 99% of the independent components in non-neural locations. Some authors also propose removal of specific muscular activity such as neck muscle activity that can affect the EEG signal during walking [22,27], by using either ICA or another blind source separation approach called canonical correlation analysis (CCA) [28]. A more general problem remains with analysis in the gamma band since gamma rhythms are generated by small volumes and are thus difficult to record with scalp EEG [29]. Moreover, the gamma band is largely contaminated by muscle activity. 

Once the EEG signal is pre-processed, two methods are mainly used for the subsequent analysis step: either ERPs if the EEG signal changes are phase-locked to an event; or time-frequency analysis in order to study induced changes (i.e., time-locked to an event) in EEG activity in different frequency bands. More recently, some groups used brain connectivity methods to study the directed and undirected functional links between different cortical areas [10], mainly during tasks involving upper limb movement [30] or cognitive tasks such as the Stroop task [31] or picture naming [11]. Some studies have also been performed during gait but in pathological conditions, mainly in patients with Parkinson’s disease [15]. 

## 3. Brain Oscillations: Principles of Time-Frequency Analysis

Non-phase-locked (induced) changes can be studied in a time-frequency analysis, which highlights the cortical oscillations related to an external or internal event [32]. EEG signal mainly represents the temporal–spatial summation of post-synaptic potentials from the local neuronal population. Oscillations in a given frequency band are the results of synchronization across neurons [33]. Indeed, motor-related cortical oscillations are generally assessed by quantifying increases (also called event-related synchronizations or ERS) or decreases (event-related desynchronizations or ERD) in spectral power in a given frequency band. Studying these oscillations across time, also called time-frequency analysis, consists in calculating the relative values of the signal power in different physiological frequency bands. The event-related spectrum (averaged over trials or related to a single trial) at each time-frequency point is either divided by the average spectral power in the pre-stimulus baseline period (during which the subject does not move) at the same frequency, or a subtraction of the average baseline power and a division by the standard deviation of the baseline power at the same frequency can be performed. These two models for the pre-stimulus baseline correction of event-related spectral perturbations (ERSPs) are respectively called gain model and additive model, and both are used in EEG studies. The units of ERSP are thus a *z*-score or a percentage of the average baseline power, but spectral perturbations can also be expressed as the log value of this percentage [34]. For example, increases in amplitude of the cortical oscillations in delta and gamma bands are observed during both the planning and execution of movement [35]. The initiation of voluntary movements has been linked to desynchronization of cortical activity in alpha and beta bands in electrocorticography and scalp EEG recordings over the motor and premotor cortices [36,37]. We should keep in mind that these oscillations, whatever the considered frequency band, are not specific to movement and have been attributed to numerous cognitive processes such as memory or attention [38,39]. Contrary to ERPs, EEG power changes do not need to be phase- or time-locked to a particular event at each trial (e.g., foot strike, start of the anticipatory postural adjustments for gait initiation). In summary, time-frequency analysis of EEG activity contributes to a better understanding of the neuronal oscillations that underlie information processing in the brain or programming of a movement. 

The most frequent pattern before and during movement, whatever its nature, is a decrease of alpha- and beta-band power starting over the sensorimotor cortex. The mu rhythm is of particular interest [40,41]. The latter is defined by activity in the alpha band recorded by scalp electrodes over the sensorimotor cortex during movement. Despite being comprised in the alpha band, the mu rhythm is distinct from alpha rhythm since the latter is recorded occipitally, reacts to eyes opening and is not specifically related to movement [42,43]. It has been firstly described that the mu rhythm reflects synchronized activity in large groupings of pyramidal neurons in the brain’s motor cortex [44]. A role of mu rhythm in the mirror neuron system [45] has then been proposed since mu rhythm is also attenuated during observed movements [46]. Despite this specificity, in most publications, it is not distinguished from the alpha rhythm. In the present review, we will use indifferently either alpha power decrease/increase (i.e., alpha ERD/ERS) or mu rhythm increase/decrease (mu ERD/ERS) according to the methodology used in the different articles. 

## 4. Cortical Oscillations during Gait Initiation in Healthy Subjects

Cortical areas involved in gait initiation include the sensorimotor cortex, premotor cortex in link with basal ganglia and brainstem structures. It was initially suggested that the motor programs underlying gait initiation were stored in subcortical structures, and could be elicited by a startling stimulus or a decision for action [1,47,48,49]. However, studies in patients with focal lesions of the supplementary motor area (SMA) and studies in patients with Parkinson’s disease have shown that the motor program can also be modulated at the supraspinal level, with implication of the SMA, the basal ganglia and the pontomedullary reticular formation [50,51]. Moreover, inhibitory repetitive transcranial magnetic stimulation over the SMA shortens the duration of anticipatory postural adjustments for a brief period, i.e., for the first stepping trial after stimulation [52]. As a consequence, cortical activation seems to directly (or via cortico-subcortical loops) modulate the timing of the motor program. The output of this pathway is located in the midbrain locomotor region (which may correspond in part to the cuneiform nucleus and the dorsal part of the pedunculopontine nucleus), which is connected to limbic structures and the basal ganglia [53].

Attentional control can also modulate gait initiation: either directly by involving brainstem structures (e.g., the alerting process induced by a loud stimulus can produce a start-react effect) or indirectly via a cortical loop that includes more complex attentional networks [48,54,55]. Indeed, gait initiation requires more attentional resources than gait [56] and may cause more dual-task interference with an attentional task than steady-state walking does [57]. For instance, errors in motor programming have been exhibited in tasks requiring executive control [16], and particularly in older subjects [58].

**Figure 3:**
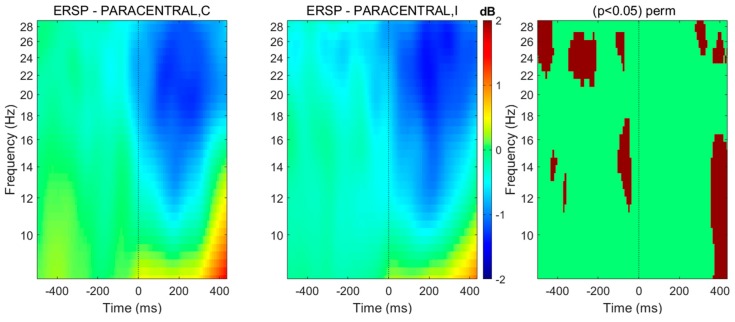
Spectral perturbations related to anticipatory postural adjustment onset located at paracentral lobules (right and left pooled), in case gait initiation is driven by a target surrounded by congruent (C) or incongruent (I) flankers. Using BRAINTSTORM toolbox [61], the head model was created from a standard MRI template and the boundary element method was used for calculating the leadfield matrix, whereas cortical sources were reconstructed thanks to weighted minimum-norm estimates before projecting the obtained dipole sources on 68 cortical regions from the Desikan-Killiany atlas. The spectral patterns were averaged over 30 healthy subjects. Spectral distribution among frequency bands from 4 to 30 Hz are shown. Right column: significant differences in a permutation (perm) test (threshold at *p* < 0.05).

It has been demonstrated that gait initiation is associated with the desynchronization of sensorimotor rhythms (alpha and beta bands) related to sensorimotor cortex activation [59]. Alpha and beta ERD are sensitive to the attentional demand, being more ample in case of selective attention required during preparation of movement. Different patterns of alpha/beta ERD during preparation of step initiation according to the attentional demand are also noticed: earlier alpha/beta ERD in case of alert stimulus, more prolonged beta ERD in case of conflicting information [16]. This implies that alpha and beta ERD during gait initiation are directly modulated by attentional abilities of the subject. This modification of sensorimotor cortex activation has direct consequences on motor commands and could lead, for example, to errors in the motor program (i.e., errors in anticipatory postural adjustments that increase with ageing). During gait, oscillations in lower bands (delta, theta) are more difficult to interpret since they largely overlap with the ERPs locked with the stimulus, leading to high inter-trial coherence (ITC; a quantification of event-related phase modulations locked to an event) [60]. We should also point out that activations over non-motor areas (prefrontal, temporal, etc.) are not specific to gait initiation but are also recorded when the attentional task is coupled, for instance, with button pressing and not with step initiation. They may reflect more adequately the attentional processes than the movement preparation itself. We can give the example of EEG scalp recordings during gait initiation of 30 healthy subjects using a flanker task in Figure 3 (signal locked to the onset of the anticipatory postural adjustments). Some of the data have been previously published in [16]. Subjects had to initiate gait with the leg indicated by the direction of a target arrow that was surrounded by either congruent or incongruent flankers. In this latter case, executive control is necessary to inhibit the incongruent flankers indicating the wrong lateralization. We observed an earlier and more ample alpha/beta ERD in case of incongruent flankers, reflecting the interaction between the executive attentional control and motor preparation. 

## 5. Cortical Oscillations during Gait in Healthy Subjects

**Figure 4:**
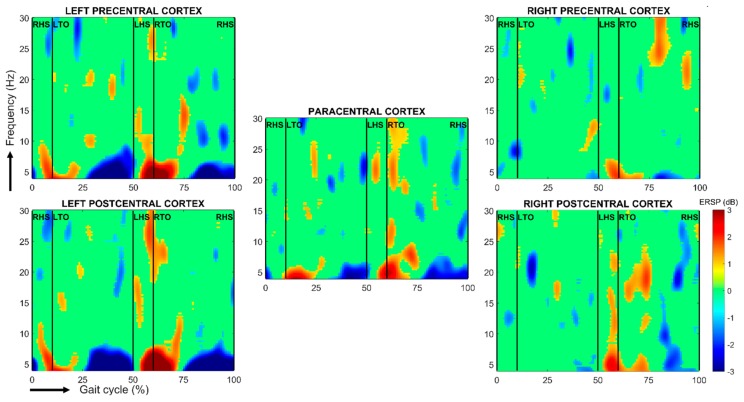
Spectral perturbations related to right heel-strike during a stride cycle relative to the full gait cycle baseline over the left and right pre-, para- and postcentral cortices. The subject performed 52 gait cycles on a treadmill. Using BRAINTSTORM toolbox [61], the head model was created from a standard MRI template and the boundary element method was used for calculating the leadfield matrix, whereas cortical sources were reconstructed thanks to weighted minimum-norm estimates before projecting the obtained dipole sources on 68 cortical regions from the Desikan-Killiany atlas. The single-trial spectrograms were linearly time-warped in order to adjust the latencies of right heel-strikes in each epoch and thus to align gait cycles. Finally, a bootstrap test was performed, with alpha = 0.1. RHS/LHS: right/left heel-strike; RTO/LTO: right/left toe-off.

The first proper analyses using methods to avoid artefacts such as ICA as described in the methodological considerations paragraph were conducted on eight subjects during treadmill walking [62]. In this analysis, each gait cycle was expressed in function of the average gait cycle. Significant alpha- and beta-band power increases over sensorimotor cortex and dorsal anterior cingulate cortex occurred during the end of stance, as the leading foot was contacting the ground and the trailing foot was pushing. This demonstrates that, even under steady-speed walking conditions, the cortex shows moment-to-moment adjustments in activity tone. In another study including six subjects walking slowly on a treadmill, the analysis consisted in expressing changes in EEG oscillations during gait according to the EEG activity during standing while fixing a cross on a computer screen [27]. When looking at changes throughout the gait cycle, a desynchronization occurred in mu and beta bands during the swing phase. Just before heel strike and during the double support phase, increases in mu and beta power were observed. These results were later confirmed ([63], robotic gait with a lokomat versus gait on treadmill) and source analysis [64] revealed that beta ERD is located in central sensorimotor area, which is consistent with somatotopic representation of leg movements [65,66]. This location found with scalp recordings was confirmed during electrocorticographic recordings of leg movements [67]. An example of a scalp EEG recording in one healthy subject by our group is given in Figure 4. Indeed, mu rhythm and beta-band power decreases observed over the central sensorimotor and parietal areas during active walking relative to standing are similar to the desynchronization in mu- and beta-band power observed in the motor system during the preparation and voluntary execution of movements [68]. Beta-band power increases in scalp EEG data are related to movement suppression or more probably to sensorimotor integration of the movement since this synchronization disappears when sensory information is disrupted [69]. Recently, electrocortigraphic recordings in two subjects pointed out the precise role of the primary motor cortex (M1) in gait pattern generation [70]. Mu, beta, and gamma oscillations were recorded during steady gait and gait across multiple walking speeds. Only gamma oscillations were consistent in both subjects during the tasks and were directly related to gait speed or gait initiation. Beta modulation was recorded in only one subject. More generally, gamma oscillations in M1 encode for high-level motor control and probably interact with subcortical/spinal networks, which are responsible for low-level motor control. The main limitation of this study was the small number of subjects and the lack of recording of key structures such as the SMA. Indeed, these results have to be compared with those from a study using fMRI and PET scan [71]. The latter study compared imagined locomotion (multiple initiations, stops, changes in speed) that recruits mainly an indirect pathway of modulatory locomotion (via the SMA, basal ganglia and mesencephalic locomotor region) and real locomotion that recruits a direct pathway of steady-state locomotion (M1 that hypothetically drives directly the central pattern generators in this model). 

Coupling between the EEG and electromyography (EMG) activity during gait has also been studied. Significant coupling between EEG recordings over the leg motor area and EMG from the anterior tibial muscle (ankle dorsal flexor) was found in the gamma frequency band prior to heel strike, during the swing phase of walking and not during the support phase [72]. In another recent study, cortical power, corticomuscular coherence, and ITC were evaluated. In contrast to the previous study, theta, alpha, beta, and gamma frequencies increased during the double support phase of the gait cycle [73]. The authors concluded that the coherent activity between M1 and muscle would reflect, due to its high ITC, an evoked response. Therefore, an additive response would be evoked during the double support phase. Recently, EEG recorded from the leg area of M1 and EMG recorded from ankle plantar flexor muscles have shown coupled gamma oscillations in the stance phase during treadmill walking [74]. Our group has also demonstrated that either excitatory or inhibitory repetitive transcranial magnetic stimulation of M1 was unable to change the level of activity of the leg muscles during gait contrary to what is observed for simple upper limb movements, suggesting a more complex role of M1 than simply controlling muscle tone during gait [75].

## 6. Cortical Oscillations during More Challenging Tasks

In daily life, subjects do not walk slowly on a treadmill but have to adapt their speed, to change their pace, to modify their stride length according to an obstacle or to navigate according to different cues (visual, auditory, etc.). When comparing walking on an incline with walking on level surface, theta power was greater in the anterior cingulate, sensorimotor and posterior parietal clusters during incline walking. It also showed differences in gamma band suggesting that these areas are implied in control of gait in these conditions [76]. Moreover, as stated earlier, cortical activations are directly linked to muscle tone although it is not the main mechanism of control [71], and corticomuscular coherence also differs according to the type of walk, predominating in swing phase during overground walking and in stance phase during ramp walking [77].

Most of the previous studies did not include gait in real-world conditions that involve cognitive processing. For example, when subjects had to adapt their speed (e.g., increasing their gait speed), beta and gamma synchronizations in prefrontal and parietal areas were enhanced, suggesting that executive control of sensorimotor areas was intensified in order to improve speed tracking performance [78]. Furthermore, according to the walking conditions (level ground, ramp ascent, and stair ascent), differences in activation in the posterior parietal cortex or M1 occurred [79]. Alpha and beta ERD was more pronounced at the beginning of gait cycle for more challenging gait conditions. Beta ERD was also larger over posterior parietal cortex.

When walking in synchrony with a series of cue tones, requiring the subject to adapt step rate and length to sudden shifts, beta-band power increases were observed in the medial prefrontal and dorsolateral prefrontal cortex [80]. Once again, the role of such cortical regions in cognitive control of gait was proposed as an explanation of this pattern and Wagner et al. [80] attributed this specific pattern of beta synchronization to cognitive top-down control. 

Dual-tasking situations are common in daily life, especially the ones which involve the concurrent performance of a cognitive task and gait [81]. For example, people often send or read a text message while walking. McIsaac et al. [82] have proposed to define dual-tasking as “the concurrent performance of two tasks that can be performed independently, measured separately and have distinct goals”. Dual-tasking can lead to changes in gait performance and these changes are considered as the costs of carrying out a second task concurrently. EEG oscillations have been evaluated during dual-tasking. In [83], four tasks were performed: normal walk on a treadmill, two dual tasks involving gait as well as additions and subtractions or video watching, and a visual-cueing task asking the subject to adapt its gait pattern. No clearly different patterns of neural oscillations in classical frequency bands were observed, although support vector machine procedures were able to classify attention tasks by differences in gamma-band activity. In another protocol replicating scenarios in which humans were required to evaluate the environment for accurate stepping (i.e., by using different color marks that forced adaptation of step length and width), Oliveira et al [84] showed changes during mid-stance in the frontal lobe and motor/sensorimotor regions, a phase in the gait cycle in which participants defined the correct foot placement for the next step. These changes consisted mainly in increases in electrocortical activity over the prefrontal cortex in beta and gamma bands when precision stepping was required. This higher neuronal synchronization in the gamma band has been related to increased attention but remains, in our opinion, difficult to isolate in scalp EEG, particularly during movement, even with the use of pre-processing methods such as ICA. 

## 7. Conclusions

As gait requires cortical resources, measuring cortical activity in real time is a necessary step to understand motor control during gait. Time-frequency analysis of EEG is adequate to record cortical activity. The main pattern of cortical activation during gait is an activation of the sensorimotor areas that is reflected by mu and beta desynchronizations predominantly during the swing phase of a gait cycle and during preparation of movement for gait initiation. Beta synchronization occurs at foot strike. Distinct roles of mu/beta oscillations are less clear than in simple movements since desynchronizations and synchronizations during gait frequently overlap both bands. Gamma oscillations are also crucial to encode gait speed or initiation, but methodological concerns still exist with scalp recordings. These spectral patterns are directly influenced by the walking context (changing the speed, overground versus ramp walking,…) and, when analysing gait with a more demanding attentional task, other areas (prefrontal, posterior parietal cortex) seem specifically involved, with the occurrence of beta/gamma oscillations. The decoding of this brain activity is a necessary step to build valid brain-computer interfaces (BCIs) able to generate gait artificially [85]. As a perspective, a real-time closed-loop BCI that decodes lower limb joint angles from scalp EEG during treadmill walking in order to control the walking movements of a virtual avatar has already been built [86]. This kind of approach could be useful in rehabilitation programs since healthy subjects are able to adapt an avatar’s gait pattern controlled via a closed-loop EEG-based BCI in eight days of training. It could be beneficial in patients suffering from either deficits in upper or lower structures of gait control such as patients with incomplete medullar injury [87], patients with stroke [88], or with Parkinson’s disease [14,89].
